# Phenotypic and proteomic analysis of positively regulated gellan biosynthesis pathway in *Sphingomonas elodea*

**DOI:** 10.1080/19768354.2017.1290678

**Published:** 2017-02-17

**Authors:** Soo Youn Lee, Ji-Young Ahn, Mihye Kim, Simranjeet Singh Sekhon, Sung-Jin Cho, Young-Chang Kim, Yang-Hoon Kim

**Affiliations:** a Climate Change Research Division, Korea Institute of Energy Research, Daejeon, South Korea; b School of Biological Sciences, Chungbuk National University, Cheongju, South Korea; c Quality Control Department, Medytox Inc., Cheongwon gu, Cheongju-si, South Korea

**Keywords:** Gellan biosynthesis, *Sphingomonas elodea*, phenotypic analysis, proteome analysis, *gel* cluster

## Abstract

*Sphingomonas elodea* is a Gram-negative bacterium capable of producing ‘gellan gum’ exopolysaccharide that is the most extensively studied expolysaccharides of microbial origin. In this study, we investigated the phenotypic and proteomic alterations in *S. elodea* by homogeneously expressing both *gelA* and *gelN* involved in positive regulation and extracellular secretion of metabolites in gellan biosynthesis, respectively. Expression of six histidine-tagged GelA and GelN was determined by Western blot analysis. Successful expression of GelA and GelN resulted in both morphological changes of colonies and enhanced secretion of gellan into the growth medium (GelA, 21.2% more and GelN, 48.3% more) overexpressed compared to the wile-type. Comparative two-dimensional gel electrophoresis analysis revealed a differential proteome expression in *S. elodea* overexpressing GelA and GelN. Proteins up- or down-regulated by GelA and GelN overexpression were found to be mainly sugar transportation proteins, two-component regulatory proteins, and proteins involved in secretion pathways. The results suggest that the effect of GelA and GelN overexpression on gellan biosynthesis might be mainly caused by increased transportation of sugar units or enhanced exportation of gellan.

## Introduction

*Sphingomonas elodea* is a Gram-negative bacterium capable of producing water-soluble and gelling agent called ‘gellan gum’ exopolysaccharide (EPS) (Kang et al. 1982). Because of its unique structure with repeating unit of heteropolysaccharides and excellent rheological characteristics, gellan gum has been used in diverse industries including the food and pharmaceutical industries (Fialho et al. 1999). The graft genome sequence of *S. elodea* ATCC 31461strain has been reported (Gai et al. 2011). Since then, much efforts have been focused on the mechanical details of gellan biosynthesis in order to improve the productivity of bacterial gellan (Zhu et al. 2011; Li et al. 2014; Osmałek et al. 2014).

**Figure 1.**
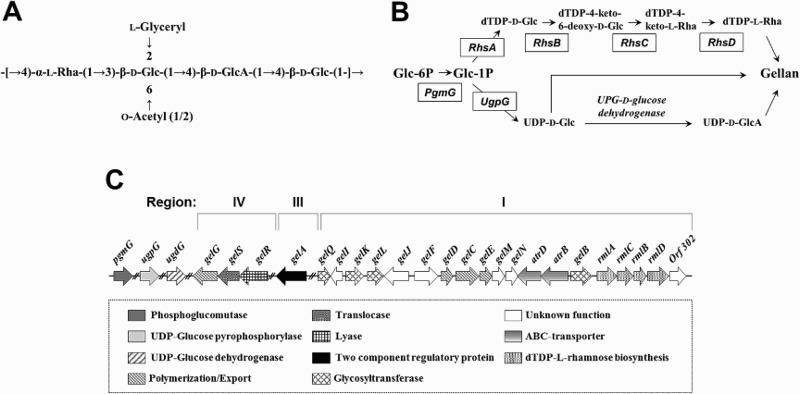
(A) Structure of the repeat unit of gellan.Glc: glucose; GlcA: glucuronic acid; Rha: l-rhamnose.(B) Gellan biosynthesis pathway in *S. elodea*. PgmgG: phosphoglucomutase; UgpG: UDP-d-glucose purophosphorylase; RhsA: dTDP-d-glucose pyrophosphorylase; RhsB: dTDP-d-glucose 4,6-dehydratase; RhsC: dTDP-4 dehydrorhamnose 3,5 epimerase; RhsD: dTDP-4-dehydrorhamnose reductase; UDP-: uridinediphospho-; TDP-: thymidine diphospho- (Sá-Correia et al. 2002). (C) Organization of the gellan biosynthetic *gel* clusters (region I and IV) and gellan region III.

The repeating unit of gellan is composed of d-glucose (d-Glc), l-rhamnose (l-Rha), and d-glucuronic acid (d-GlcA). It forms the linear heteropolysaccharide structure as tetrasaccharide (Figure 1(A)) (Jansson et al. 1983; Sá-Correia et al. 2002). Enzymes involved or predicted to be involved in gellan biosynthesis pathway are two metabolic intersections by UDP-d-glucose pyrophosphorylase (UgpG) and dTDP-d-glucose 4,6-pyrophosphorylase (RhsA) (Figure 1(B)) (Sá-Correia et al. 2002). Consequentially, gellan precursors UDP-d-glucose, UDP-d-glucuronic acid and dTDP-l-rhamnose are colocalized and assembled by genes involved in the *gel* cluster (Schmid et al. 2014).

As shown in Figure 1(C), *gel* clusters comprise at least 20 genes coding for enzymes involved in the synthesis of dTDP-l-rhamnose (*rmlABCD*), specific glycosyltransferases, and protein required for gellan polymerization and export (Harding et al. 2004).The *gel* cluster is categorized in to three regions (regions I, III, and IV). Region I is involved in the assembly of tetrasaccharide repeat-units by *gelQ, gelK, gelL* and *gelB*. Region I and IV are involved in gel polymerization and export by *gelS, gelG, gelC, gelE,* and *geld.* Region III includes two component regulatory protein *gelA* (Fialho et al. 2008). The *gelA*, encoding a two-component regulatory protein, is presumed to turn on gellan biosynthesis depending on environmental conditions that bacteria are exposed due to its regulatory cascade (Harding et al. 2004). Downstream components in gellan biosynthesis pathway might be directly affected by the expression level of *gelA* (Garg et al. 2000). In addition, *gelN* coding enzyme has been predicted to be involved in protein export (namely proteins targeted to the periplasm or outer membrane) by comparative sequence analysis between *S. elodea* ATCC 31641 and *Sphingomonad* sp. ATCC 31554 (S-88 strain, *sps* cluster) (Yamazaki et al. 1996; Haft et al. 2006; Fialho et al. 2008). Therefore, changes in intracellular expression of *gelA* and *gelN* gene might trigger the regulation of gellan biosynthesis, thus altering global responses in targeted metabolic pathway.

The objective of this study was to investigate the responses of *S. elodea* strain overexpressed with two pivotal genes (*gelA* and *gelN*) involved in gellan biosynthesis and secretion. First, *gelA* and *gelN* genes were cloned into broad-host-range vector pBBR122 and expressed homogeneously in *S. elodea*. Phenotypic changes of this bacterium were compared by quantifying the amount of gellan produced with morphological observations. Moreover, comparative proteomic analysis was performed between wild-type *S. elodea* and *S. elodea* everexpressing *gelA* and *gelN* to determine the effect of everexpression of *gelA* and *gelN* on gellan biosynthesis. This might shed light on the molecular mechanisms underlying the regulation of gellan biosynthesis and the target metabolic.

## Materials and methods

### Bacterial strains and culture conditions

Bacterial strains and plasmids used in this study are summarized in Table 1. Wild-type *S. elodea* ATCC 31461 was cultivated in Luria-Bertani (LB) medium [1% (w/v) tryptone, 0.5% (w/v) yeast extract, 0.5% (w/w) NaCl] at 30°C for 72 h. *Escherichia coli* BL21 (DE3) strain was used as host for genetic manipulation and cultured in LB medium at 37°C. LB solid medium contained 15 g L^−1^ agar. For gellan production, one loop full of *S. elodea* slant culture was transferred to 50 mL of LB medium in Erlenmeyer flasks. Flasks were incubated at 30°C in a rotary shaker (150 rpm) for 24 h for inoculum development. Then 1 mL of the broth was used as inoculum for 100 mL of S-medium [10 g Na_2_HPO_4_, 3 g KH_2_PO_4_, 1 g K_2_SO_4_, 0.2 g MgSO_4_•7H_2_O, 10 mg CaCl_2_, 1 mg FeSO_4_•7H_2_O, 1 g casamino acid, 1 g yeast extract, and 20 g glucose per 1 L of distilled water] in 500 mL of Erlenmeyer flasks followed by incubation at 30°C in a rotary shaker (150 rpm) for 24–72 h. When necessary, antibiotics were used at the following concentrations: 25 mg L^−1^ streptomycin for *S. elodea*, 25 mg L^−1^ ampicillin for *E. coli* strains transformed with pET-21a and 50 mg L^−1^ kanamycin for *S. elodea* strains transformed with pBBR122. Culture growth in flasks was monitored by measuring the optical density at 640 nm (OD_600_) using a UV/Vis spectrophotometer (Mecasys Co., Ltd., Korea).

**Table 1.** Bacterial strains and plasmids used in this study.

Bacterial strains and plasmids	Relevant characteristics	Source or reference
*Bacterial strains*		
*S. elodea*	Str^r^	ATCC 31461^a^
*E. coli* BL21 (DE3)	F^-^*ompThsdS_B_*(*r_B_^-^m_B_^-^*) *galdcm*(DE3)	Novagen, Germany
Plasmids		
pET-21a	Ap^r^, T7 *lac* promoter, f1 origin; c-terminal six histidyl fusion vector	Novagen, Germany
pEGA	pET-21a carrying the *gelA* structural gene	This work
pEGN	pET-21a carrying the *gelN* structural gene	This work
pBBR122	Cm^r^, Km^r^, linearized at the unique *Eco*72 I site and dephosphorylated	MoBiTec, Germany
pBGA	pBRR122 carrying the *gelA-His*_6_	This work
pBGN	pBRR122 carrying the *gelN-His*_6_	This work

^a^American Type Culture Collection

### Genetic manipulations

Genomic DNA extractions, plasmid preparations, PCR reactions, ligations, transformations, and other standard molecular biology techniques were carried out as described elsewhere (Sambrook & Russell 2012) or following the instructions of the supplier. PCR experiments were carried out by using T Gradient thermocycler (Biometra, Germany) and Ex Tag DNA polymerase (Takara Bio, Inc., Japan). Genomic DNA extracted from *S. elodea* was used as the template. PCR products were purified using QIAquick PCR purification kit (Qiagen, Germany). *E. coli* and *S. elodea* cells were transformed by electroporation using Electro Cell Manipulator (BTX Technologies Inc., USA).

### Construction of recombinant plasmids

All oligonucleotide sequences used in this study are listed in Table 2. Coding regions of *gelA* and *gelN* were amplified from the genomic DNA of *S. elodea* by PCR. Primer pairs gelA-F/gelA-R were used for the amplification of *gelA* gene and gelN-F/gelN-R primers were used for the amplification of *gelN* gene. PCR products of *gelA* and *gelN* genes were digested with *Eco*RI/*Hind*III and ligated with plasmid pET-21a digested with the same restriction enzymes. The resulting plasmids named ‘pEGA’ and ‘pEGN’, respectively, were transformed into *E. coli* BL21 (DE3). Subsequently, *gelA* and *gelN* coding regions with N-terminal six histidine (His_6_) tag sequence were amplified from pEGA and pEGN plasmids using primer pairs gelA-His-F/gelAN-His-R and gelN-His-F/gelAN-His-R, respectively. These secondary PCR products were digested with *Acc*III/*Sau*I for *gelA*-His_6_ and *Age*I/*Sau*I for *gelN*-His_6_ and ligated with plasmid pBBR122 digested with restriction enzymes of *Acc*III/*Bau*36I and *Age*I/*Bau*36I, respectively. The resulting plasmids named ‘pBGA’ and ‘pBGN’, respectively, were transformed into *S. elodea*.

**Table 2.** Primers used in this study.

Primers	Sequences (5′−3′)^a^	Restriction enzyme sites
gelA-F	TAGG/AATTCAGGCAGCATTGGCGTA	*EcoR*I
gelA-R	TAGA/AGCTTTTTGGCCACCAGGATC	*Hind*III
gelN-F	GCGG/AATTCCGACTATCCGATCCTG	*EcoR*I
gelN-R	TAGA/AGCTTGGCGCGCTCGGCGCGC	*Hind*III
gelA-His-F	T/CCGGAAGATCCCGATCCCGCGAAA	*Acc*III
gelN-His-F	TATA/CCGGTAGATCTCGATCCCGCGAAA	*Age*I
gelAN-His-R	TAGCC/TCAGGCAAAAAACCCCTCAAGAC	*Sau*I^b^

^a^Underline indicates restriction enzyme site for cloning with pET-21c or pBBR122.

^b^*Sau*I restriction enzyme site,CC/TNAGG (isoschizomer enzyme with *Bau*36I).

### Homogeneous expression of GelA and GelN proteins

Sodium dodecyl sulfate-polyacrylamide gel electrophoresis (SDS-PAGE) and western blot analyses were used to analyze proteins of *E. coli* and *S. elodea* strains using standard protocols (Sambrook & Russell 2012). Western blot analyses of GelA and GelN protein were performed using His-Tag monoclonal antibody (Novagen, Madison, WI, USA).

### Quantification of extracellular polysaccharides

Cultures were centrifuged at 13,000 rpm for 30 min at 25°C. One volume of cell-free supernatant was added to three volumes of 95% ethanol (v/v) to precipitate gellan gum. The precipitate was recovered by centrifugation at 13,000 rpm for 10 min at 25°C and dried in a hot-air oven (60°C) for 24 h. The precipitate was stained with Alcian blue (1 g FlukaAlcian Blue 8GS with 100 mL of 18 mM H_2_SO_4_) and incubated at 37°C for 30 min. At the end of incubation, the optical density of the sample was measured wavelength at 677 nm. The concentration of gellan was assayed using Alcian Blue dye stock solutions consisted of 1, 1/10, 1/100, and 1/1000 dilutions of the original dye solution. The concentration of glucose in the broth medium was assayed using a Glucose Assay Kit (Sigma-Aldrich) according to the manufacturer’s protocol. All experimental data given below are mean values obtained from three independent determinations.

### Sample preparation for proteome analysis

*S. elodea* cells were grown in 100 mL of S-media until late stationary-phase (72 h). They were harvested by centrifugation at 6000 rpm for 20 min at 4°C. Cell pellets were washed three times in an equal volume of ice-cold 0.2 M sucrose followed by washing with 5 mL ice-cold methanol. Cell pellets were resuspended in 10 mM Tris-EDTA buffer (pH 8.0) and disrupted by sonication. Cell debris in samples was moved by centrifugation at 13,000 rpm for 20 min at 4°C. To remove nucleic acids, 0.2% (v/v) DNase I (5 U μL^−1^) and 0.2% (v/v) RNase (10 mg mL^−1^) were added to each sample and incubated on ice for 30 min. After adding 100% ice-cold acetone and 100% trichloroacetic acid (1:8:1 ratio) followed by incubation at −20°C for 20 min, cell pellets were precipitated by centrifugation at 13,000 rpm for 20 min at 4°C. The supernatant was decanted after the centrifugation. Pellets were washed with 1 mL ice-cold acetone and centrifuged again at the same condition. The supernatant was decanted and protein pellets were resuspended in 1 mL of lysis buffer [1 M Urea, 2 M Thiourea, 4% CHAPS, 40 mM Tris, Protease inhibitor cocktail] and mixed vigorously. Protein concentration was determined using 2-D Quant Kit (Amersham Biosciences, Sweden). Protein samples were then stored at −70°C.

### Two-dimensional gel electrophoresis and image analysis

A total of 60 μg protein was resuspended in 350 μL rehydration solution [7 M Urea, 2 M Thiourea, 4% CHAPS, 20 mMtris, 0.5% IPG buffer (Amersham Bioscience)], 200 mM TBP and 0.02% bromophenol blue. Protein samples were applied to 18 cm-length (pH 4-7) IPG Dry Strips (Amersham Biosciences) followed by rehydration at 20°C for 12 h. Isoelectric focusing was conducted using an EttanIPGphor II (Amersham Biosciences) at a constant temperature of 20°C with a total of 35,500 Vh as follows: 500 V for 1 h; 1000 V for 1 h; 8000 V until 32,000 Vh was reached. IPG strips were incubated with equilibration solution [6 M urea, 50 mMtris-Cl (pH 8.8), 30% (v/v) glycerol, 2% SDS, 5 mM TBP, 0.02% bromophenol blue] for 15 min at room temperature with gentle shaking. SDS PAGE was performed in PROTEAN II xi Cell (Bio-Rad, Hercules, CA, USA) using 12% SDS-polyacrylamide gels (20 × 20 cm) at a constant temperature of 20°C under 200 V, 200 mA for 6 h.

### Image analysis

To visualize protein electrophoresis, silver staining was carried out using PlusOne Silver Staining Kit (Amersham biosciences). Stained-2DE gels were scanned using PowerLook 1100 (UMAX, Korea). Comparative image analysis was carried out using Progenesis software (Nonlinear Dynamics, Newcastle, UK). Gels were stained with silver to select spots of interest. Meanwhile, Coomassie brilliant blue staining was performed for spot identification by mass spectrometry. For protein identification, proteins in gel were fixed by soaking in a solution containing 40% (v/v) methanol and 10% (v/v) acetic acid for 1 h with gentle agitation. Fixed gels were stained by colloidal Coomassie staining solution (ProteomeTechInc, Korea).

### MALDI-TOF mass spectrometric analysis and protein identification

Enzymatic digestions were performed overnight at 37°C in a stationary incubator using 10 – 15 μg mL^−1^of sequencing-grade modified trypsin (Proma, Wisconsin, USA) in 25 mM ammonium bicarbonate (pH 8.0). In-gel-digested peptide fragments were extracted from gel pieces using a solution containing 5% (v/v) trifluoroacetic acid (TFA) in 50% (v/v) acetonitrile (ACN) by vortexing for 1 h. After repeating this extraction procedure three times, solute materials including peptide fragments were dried using vacuum evaporator. The peptide solution was prepared with an equal volume of saturated *α*-cyano-4-hydroxy-cinnamic acid solution in 50% ACN/0.1% TFA on a sample plate of AMLDI-TOF mass spectrometer. Protein analyses were performed using MALDI-TOF mass spectrometry system (Voyager DE-STR, Applied Biosystems). Spectra were calibrated using matrix and Tryptic autodigestion ion peaks were used as internal standards. Peptide mass fingerprints were analyzed using MASCOT peptide fingerprint supported by Matrix Science (http://www.matrixscience.com/). The identification of protein with respective theoretical parameters (pI, molecular mass) was accepted if the peptide mass matched with a mass tolerance within 10 ppm.

## Results

### Homogeneous expression of GelA and GelN in *S. elodea*

**Figure 2.**
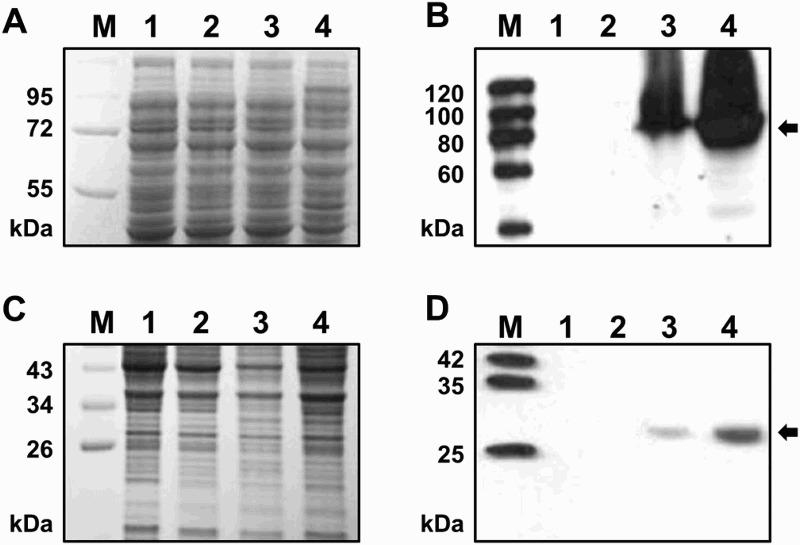
SDS-PAGE (A, C) and western blot analysis (B, D) results of homogenous expression of *gelA* (A, B) and *gelN* (C, D) gene in *S. elodea*. Lane M: protein size marker; lane 1: whole proteins of wild-type *S. elodea* (72 h); lane 2: whole proteins of *S. elodea* transformed with pBBR122 (72 h); lane 3: whole proteins of *S. elodea* transformed with pBBR122 carrying *gelA* (pBGA); lane 4: whole proteins of *S. elodea* transformed with pBBR122 carrying *gelN* (pBGB); lane 3: protein samples were prepared from mid-loge phase cultures of each cell (24 h); lane 4: protein samples were prepared from stationary phase cultures of each cell (72 h).

The lengths of *gelA* and *gelN* structural gene of *S. elodea* are 2391 bp and 699 bp, respectively. Recombinant plasmids pBGA and pBGN (pBBR122 carrying each *gelA* and *gelN* structural gene with His_6_-tag) were transformed and expressed in wild-type *S. elodea* strain as described earlier. SDS-PAGE and Western blotting results of homogenous expression of His_6_-tagged GelA and GelN in *S. elodea* are shown in Figure 2. Since both proteins belong to a family of integral membrane proteins, excessive expression might be harmful to the cells (Shin et al. 2010; Haft et al. 2012). Therefore, pBBR122-based low-copy-number recombinant plasmids (pBGA and pBGN) were more appropriate for their expression. Successful expression of GelA-His_6_ and GelN-His_6_ were clearly observed based on western blot (Figure 2(B) and 2(D)). Protein bands corresponding to their expected sizes (88.9 kDa for GelA-His_6_ and 26.8 kDa for GelN-His_6_) were observed from cell lysates carrying the two recombinant plasmids (lanes 3 and 4). Such bands were absent from cell lysate without the vector (lane 1) or carrying an empty vector (lane 2). The amounts of expression products of GelA-His_6_ and GelN-His_6_ after 72 h cultivation (lane 4, Figure 2(B) and 2(D)) were increased significantly (up to1.5 folds and 4.8 folds, respectively) compared to products after 24 h cultivation (lane 3).

### Phenotypic changes and gellan biosynthesis in *S. elodea*

**Figure 3.**
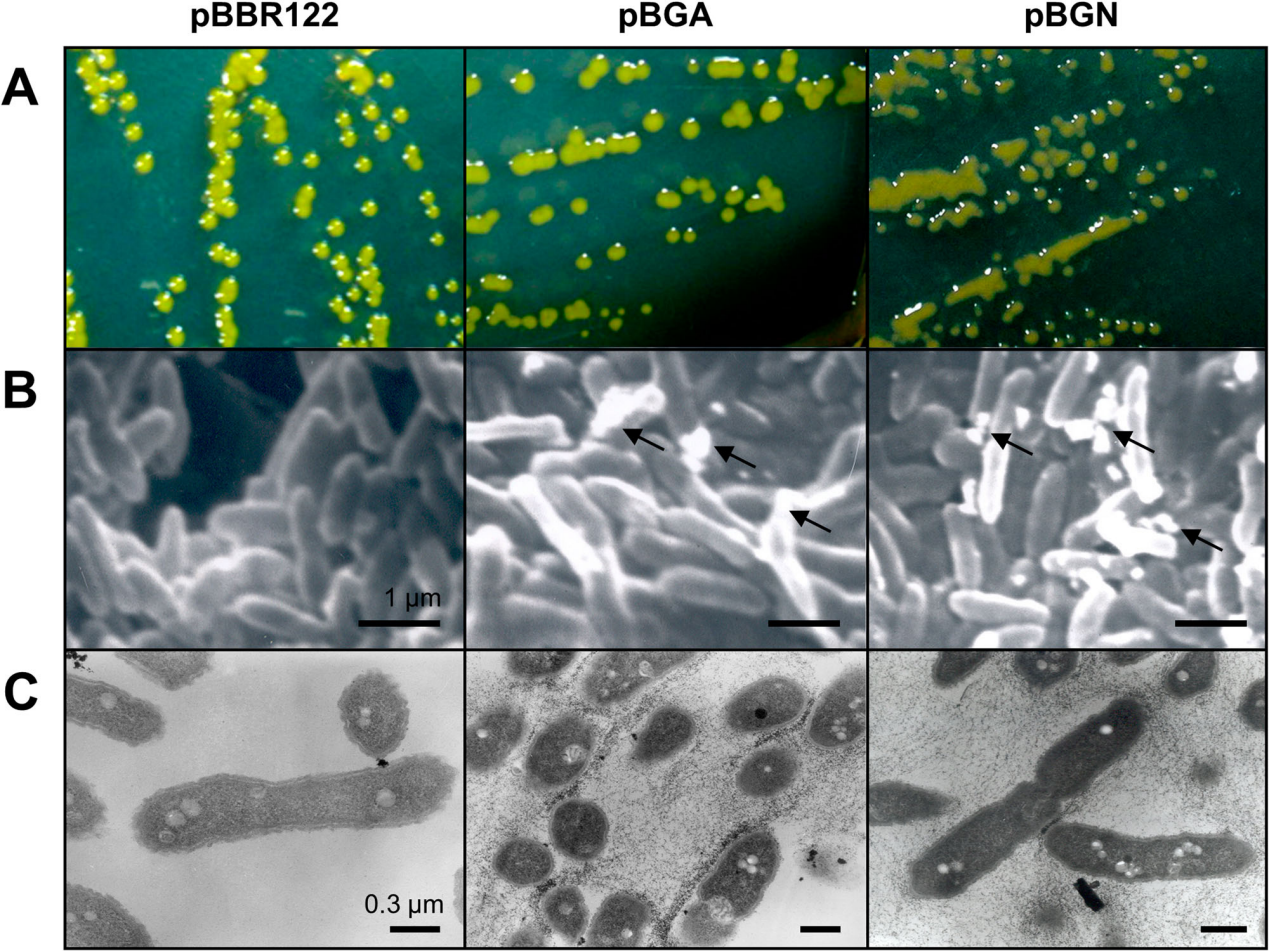
Phenotype comparison of empty pBBR122 (control) and, pBGA (*gelA*) of pBGN (*gelN*) transformed *S. elodea*. (A) Colony morphologies (72 h of cultivation on S-media plate at 30°C) of three-types of *S. elodea* strains. (B) SEM and (C) TEM images of cultured cells in liquid S-media for 72 h at 30°C, 150 rpm. Arrows indicate extracellular gellan lumps.

Homogenous and overexpression of GelA and GelN affected the phenotype of *S. elodea*. As shown in Figure 3(A), colony morphologies of the two *S. elodea* strains transformed with pBGA and pBGN were more viscous than the wild-type strain. Scanning electron microscopy (SEM) and transmission electron microscopy (TEM) analyses revealed that saccharide-like lumps or precipitates were released around the extracellular surfaces of these bacteria (Figure 3(B) and 3(C)). The morphologic changes of *S. elodea* strains resulted in enhanced gellan biosynthesis as expected (Table 3). Cell growth, glucose consumption, and gellan production were observed for different *S. elodea* strains grown in S-media containing 20 mg mL^−1^ glucose as carbon source. There was no significant difference in bacterial growth. However, the amount of extracellular gellan was enhanced by *S. elodea* with homologous expression of GelA (21.2%) and GelN (48.3%) compared to *S. elodea* strain carrying empty pBBR122.

**Table 3.** Quantification of residual glucose and extracellular gellan concentrations during cultivation of *S. elodea* strains.

	Cell growth (OD_640_)			Residual glucose (mg mL^−1^)^a^			Gellan (mg mL^−1^)		
	24 h	48 h	72 h	24 h	48 h	72 h	24 h	48 h	72 h
*S. elodea*	1.8 ± 0.1	2.3 ± 0.1	2.4 ± 0.2	16.2 ± 0.2	13.2 ± 0.5	10.5 ± 0.9	4.8 ± 1.2	7.3 ± 1.7	12.3 ± 0.8
*S. elodea* (pBBR122)	1.6 ± 0.2	2.1 ± 0.2	2.3 ± 0.1	16.8 ± 0.3	13.3 ± 0.8	10.9 ± 0.0	3.2 ± 1.0	6.8 ± 0.5	11.8 ± 1.1
*S. elodea* (pBGA)	1.3 ± 0.5	1.6 ± 0.7	1.9 ± 0.6	18.2 ± 0.8	13.0 ± 0.5	8.4 ± 0.2	2.5 ± 0.7	6.6 ± 1.1	14.3 ± 1.0
*S. elodea* (pBGN)	1.9 ± 0.3	2.4 ± 0.4	2.5 ± 0.4	16.5 ± 0.7	12.9 ± 1.0	7.9 ± 0.3	2.4 ± 0.9	6.7 ± 0.8	17.5 ± 1.2

^a^Initial glucose concentration was 20 mg mL^−1^.

### Proteomic responses in *S. elodea* to expression of GelA and GelN

**Figure 4.**
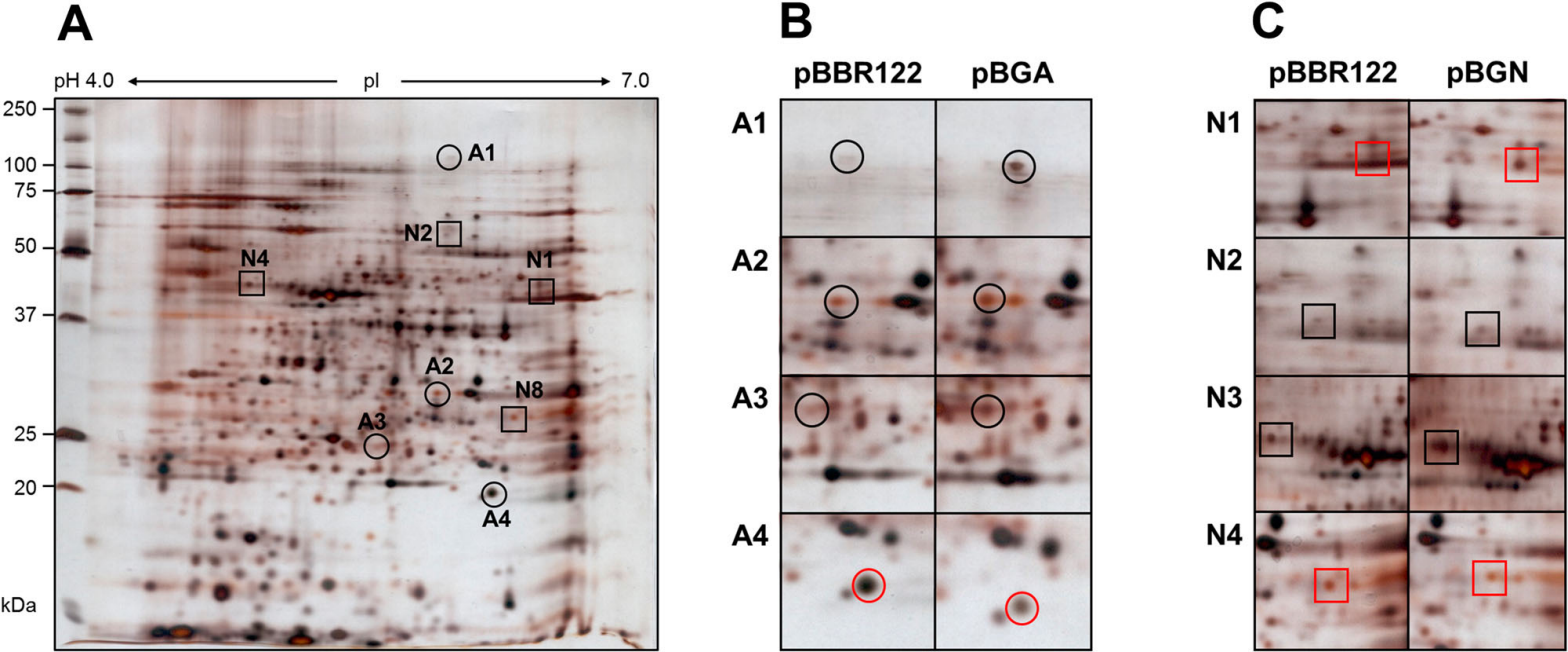
Protein expression profile of (A) empty pBBR122 transformed *S. elodea*. Circles (spot A1 to A4) indicate significantly changed spot intensities comparing to *gelA*-carrying pBBR122 (pBGA) transformed *S. elodea* strain. Squares (spot N1 to N4) indicate significantly changed spot intensities comparing to *gelN*-carrying pBBR122 (pBGN) transformed *S. elodea* strain. The spot numbers in (B) and (C) correspond to those in (A). Black color indicates up-regulated protein spots. Red color indicates down-regulated protein spots by homogenous expression of *gelA* or *gelN* gene in *S. elodea*.

To determine the influence of overexpression of GelA and GelN proteins on proteomic profiled of *S. elodea*, two-dimensional gel electrophoresis (2-DE) experiments of *S. elodea* strains transformed with pBGA and pBGN were performed. Results are shown in Figure 4. After 72 h cultivation, total intracellular proteins were prepared from cultured after disrupting cell pellets. For each protein sample, 2-DE was repeated at least five times. After silver staining, average 2-DE image was used for comparative image analysis. Figure 4(A) shows protein expression profile of *S. elodea* transformed with empty pBBR122 vector as a control. Through comparative image analysis, overexpression of GelA resulted in four differentially expressed protein spots (spot A1 to A4, Figure 4(B)) compared to control. Similarly, overexpression of GelN resulted in four differentially expressed protein spots (spot N1 to N4, Figure 4(C)). These eight protein spots were subjected to peptide fragmentation. Results of MALDI-TOF mass spectrometric analysis and protein identification are shown in Table 4.

**Table 4.** Results of MALDI-TOF mass spectrometric analysis and protein identification for the eight protein spots of *S. elodea* transformed with pBGA or pBGN showing significant differences in intracellular protein level.

No.	Gene ID^a^	Protein name	pI	MW (kDa)	Accession number^b^	% Sequence coverage^c^	Changes in spot intensity^d^
A1	Becp1808	EPS transport protein in *Burkholderiavietnamiensis* G4	6.52	93.2	ABO59421	41	+3.61
A2	BPSL1793	Putative sugar-binding exported protein in *Burkholderiapseudomallei*	5.99	32.6	CAH35792	50	+2.57
A3	MED92_00345	Two-component system regulatory protein in *Neptuniibactercaesariensis*	5.32	26.03	EAR60333	64	+2.11
A4	BAV1250	Putative sugar epimerase/dehydratase in *Bordetellaavium* 197N	6.54	38.27	CAJ48858	76	−2.10
N1	Xaut_3131	UDP-n-acetylglucosamine 1-carboxyvinyltransferase in *Xanthobacter*sp. Py2	6.12	45.41	ABS68361	51	−3.81
N2	BTH_I0522	Mannose-1-phosphate guanylyltransferase/ mannose-6-phosphate isomerase in *Burkholderiathailandeosis*	5.50	57.38	ABC38076	48	+2.70
N3	Mlg_2385	General secretion pathway protein L in *Alkalilimicolaethrlichei* MLHE-1	5.05	46.06	ABI57725	58	+3.23
N4	MXAN_1944	Glycosyltransferase, group 2 family protein in *Mycococcusxanthus* DK1622	6.38	26.42	ABF91903	59	−3.53

^a^Gene name or locus_tag from NCBI reference sequences.

^b^Accession code refers to the SWISS-2DPAGE database.

^c^Mass tolerance in protein identification through PMF experiments was 10 ppm.

^d^Protein expression levels were compared with wild-type *S. elodea.*

As shown in Figure 4(B), overexpression of GelA resulted in up-regulation of EPS transport protein (spot A1), sugar-binding exported protein (spot A2), and two-component system regulatory protein (spot A3). On the other hand, sugar epimerase-related protein (spot A4) was down-regulated as a result of GelA overexpression. Epimerization of sugar unit might have altered the physicochemical properties of the polymer, thus affecting bacterial pathogenicity, virulence, and environmental adaptability (Whitfield et al. 2015). 2-DE results of *S. elodea* (pBGN) (Figure 4(C)) showed that two protein spots, mannose-1-phosphate guanylyltransferase/mannose-6-phosphate isomerase (spot N2) and general secretion pathway protein L (spot N3), were up-regulated as a results of overexpression of GelN. The identified proteins are related to the transfer of sugar monomer (i.e. mannose) to guanosine triphosphate (GTP) and extracellular secretion (Sandkvist et al. 1999; Zakrzewska et al. 2003). Intracellular glycosylation might have been activated by GelN overexpression, resulting in enhanced secretion in *S. elodea*. Meanwhile, UDP-a-acetylglucosamine 1-carbosyvinyltreansferase (N1) and glycosyltransferasegroup family 2 protein (N4), two proteins related to cell wall biosynthesis, were down-regulated by GelN overexpression (Wanke et al. 1992; Goldman et al. 2006).

## Discussion

Over the last decade, EPS synthesized by microbes have received great interest with possibility to replace fossil based polymers (Rehm 2010). Gellan is one of the most extensively studied EPS of microbial origin due to its advantageous properties such as biodegradability, non-toxicity, rapid gelation in the presence of cations, high water holding capacity, and mucoadhesive potential (Osmałek et al. 2014).

Recently, the draft genome sequence of *S. elodea* ATCC 31461 has been revealed using Illumina paired-end technology and Velvet software (Gai et al. 2011). At least 42 enzymes related to metabolism of monosaccharides such as mannose, d-galactonate, d-gluconate, ketogluconates, d-galactonate and d-glucuronate have been predicted (Gai et al. 2011). However, genes involved in the biosynthesis, regulation, and modification of gellan have not been fully characterized. The process of gellan biosynthesis follows several steps: (i) nucleotide precursors, (ii) formation of tetrasaccharide repeating unit, (iii) backbone modification, and (iv) polymerization and export (Harding et al. 2004). Deep understanding of gellan biosynthesis pathway and regulatory mechanism is crucial to enhance the productivity and create tailor-made polysaccharide variants by genetic engineering (Schmid et al. 2014).

In this study, we reported the cloning of *gelA* and *gelN* genes from gellan-producing bacterium *S. elodea* ATCC 31461 and homogeneously expressed them in order to investigate global cellular responses (Figure 2). GelA has been predicted to a two-component regulatory protein (positive regulator), not linked to the main gellan biosynthetic gene cluster (Harding et al. 2004). In addition, Harding et al. (2004) have reported that mutant *S. elodea* deleted with *gelN* gene has reduced gellan productivity compared to its wild-type strain. The effect of overexpression of GelA and GelN on gellan biosynthesis might be due to increased transportation of sugar units or enhanced exportation of gellan.

Phenotypic analysis of this study revealed that overexpression of both GelA and GelN changed colony morphologies due to enhanced gellan production and release compared to wild-type and control (empty-vector contained) strains (Figure 3 and Table 3). Proteomic analysis results suggest that GelA and GelN might have affected biosynthesis pathways related to EPS biosynthesis and exportation (Figure 4 and Table 4). However, these results showed little information about the functional characteristics of gellan biosynthetic pathways. Further studies are needed to determine the regulatory roles and mechanisms of *gel* cluster to provide additional useful information to help elucidate the mechanisms involved in gellan gum biosynthesis in *S. elodea*.
